# Molecular Mechanisms of Proteinuria in Minimal Change Disease

**DOI:** 10.3389/fmed.2021.761600

**Published:** 2021-12-23

**Authors:** Shrey Purohit, Federica Piani, Flor A. Ordoñez, Carmen de Lucas-Collantes, Colin Bauer, Gabriel Cara-Fuentes

**Affiliations:** ^1^Division of Renal Diseases and Hypertension, Department of Medicine, University of Colorado Anschutz Medical Campus, Aurora, CO, United States; ^2^Department of Pediatrics, Section of Pediatric Nephrology, Children's Hospital Colorado, Aurora, CO, United States; ^3^Department of Medicine and Surgery Sciences, Alma Mater Studiorum University of Bologna, Bologna, Italy; ^4^Division of Pediatric Nephrology, Hospital Universitario Central de Asturias, Oviedo, Spain; ^5^Division of Pediatric Nephrology, Hospital Niño Jesus, Madrid, Spain

**Keywords:** minimal change disease, nephrotic syndrome, proteinuria, immune cell, podocyte, circulating factor

## Abstract

Minimal change disease (MCD) is the most common type of idiopathic nephrotic syndrome in childhood and represents about 15% cases in adults. It is characterized by massive proteinuria, edema, hypoalbuminemia, and podocyte foot process effacement on electron microscopy. Clinical and experimental studies have shown an association between MCD and immune dysregulation. Given the lack of inflammatory changes or immunocomplex deposits in the kidney tissue, MCD has been traditionally thought to be mediated by an unknown circulating factor(s), probably released by T cells that directly target podocytes leading to podocyte ultrastructural changes and proteinuria. Not surprisingly, research efforts have focused on the role of T cells and podocytes in the disease process. Nevertheless, the pathogenesis of the disease remains a mystery. More recently, B cells have been postulated as an important player in the disease either by activating T cells or by releasing circulating autoantibodies against podocyte targets. There are also few reports of endothelial injury in MCD, but whether glomerular endothelial cells play a role in the disease remains unexplored. Genome-wide association studies are providing insights into the genetic susceptibility to develop the disease and found a link between MCD and certain human haplotype antigen variants. Altogether, these findings emphasize the complex interplay between the immune system, glomerular cells, and the genome, raising the possibility of distinct underlying triggers and/or mechanisms of proteinuria among patients with MCD. The heterogeneity of the disease and the lack of good animal models of MCD remain major obstacles in the understanding of MCD. In this study, we will review the most relevant candidate mediators and mechanisms of proteinuria involved in MCD and the current models of MCD-like injury.

## Introduction

Minimal change disease (MCD) is the most common type of nephrotic syndrome in children, whereas it only accounts for 10–16% cases in adults (1, 2). The term MCD refers to a histological pattern characterized by the normal or near-normal appearance of glomeruli on light microscopy and immunofluorescence with podocyte foot process effacement (FPE) on electron microscopy as the sole abnormality observed in kidney biopsy (3). While histological findings are similar in children and adults with MCD, the clinical response to steroids, considered as first-line therapy, is different. Most children achieve resolution of proteinuria within days, whereas it can take months in adults (4, 5). Therefore, a kidney biopsy is only performed in selected pediatric cases, whereas it is mandated in all the adults to rule out other forms of nephrotic syndrome including infections, malignancies, or other glomerular diseases. For children who respond to steroid therapy, namely steroid-sensitive nephrotic syndrome (SSNS), MCD represents the most common underlying histological pattern followed by focal segmental glomerulosclerosis (FSGS), a more severe form of nephrotic syndrome involving glomerular scarring (4).

The name MCD can be misleading. While kidney histology shows minor changes and MCD has been traditionally considered a benign disease, it is often associated to multiple relapses, important comorbidities, and serious complications [acute kidney injury (AKI), thrombotic disorders, infections, etc.] (6–8). In addition, the pediatric onset disease can persist in adulthood (9). Therefore, MCD represents an important burden to patients, families, and the healthcare system (10). Some patients also develop resistance to therapies and/or progression toward advanced stages of chronic kidney disease (CKD) and this is usually associated with a change in the glomerular histology from MCD to FSGS (11, 12). So, a diagnosis of MCD may not be definitive; but, whether MCD and FSGS are distinct diseases or a continuum of the same disease remains unclear (13).

**Figure 1:**
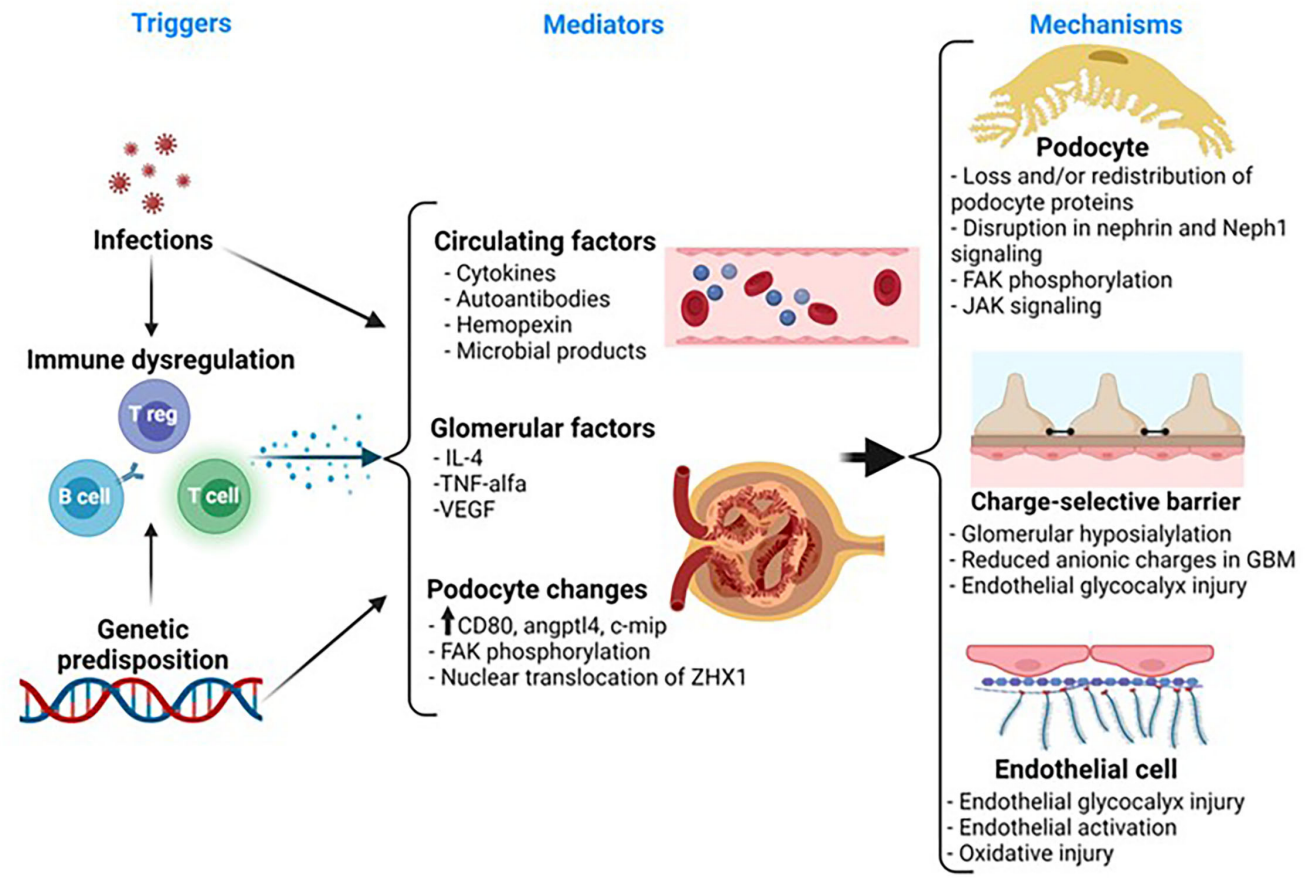
Representative schematic of the pathogenesis in MCD. MCD is associated with immune dysregulation. Notably, there is a strong association between human leukocyte antigen (HLA) and susceptibility to idiopathic nephrotic syndrome. So, it is possible that children with MCD have a genetic predisposition toward immune dysregulation that, in turn, contributes to release of factors that may play a role in the pathogenesis of the disease. Also, infections often trigger relapse in these patients and this may be the result of an exaggerated immune response in a susceptible patient. Several candidate mediators, released by systemic T or B cells or by glomerular cells, have been suggested to play a role in the development of proteinuria in MCD, but to date, there is a no definitive mediator of MCD. The podocyte exhibits morphological and molecular changes involving key proteins such as synaptopodin, FAK, and nephrin, but these changes are not specific for MCD and the upstream pathways leading to these changes remain unknown. The glomerular basement membrane (GBM) and the endothelial cell also show subtle changes. Thus, there is a loss of anionic charges in the GBM, but this does not appear to be a key driver of proteinuria. More recently, there is evidence of endothelial cell activation and oxidative stress, but whether this may play a role in the disease or it may represent a paraphenomenon is unclear. MCD, minimal change disease; IL, interleukin; VEGF, vascular endothelial growth factor; Angptl4, angiopoietin-like 4; ZHX1, zinc fingers and homeoboxes; FAK, focal adhesion kinase; JAK, Janus kinase.

The clinical hallmark of MCD is sudden-onset proteinuria and anasarca. However, the mechanisms of proteinuria remain poorly understood (14). The lack of inflammatory cells and immune complexes in the MCD glomerulus led to the hypothesis that some circulating factor(s), presumably released by T cells, may trigger proteinuria and podocyte injury (15–18). This assumption is widely accepted by the nephrology community and it is supported by some clinical observations; but the presence, nature, and the cell source for the presumed circulating factor(s) have remained elusive for decades. With the discovery of nephrin as a key protein in the podocyte slit diaphragm (SD) and the glomerular filtration barrier (GFB), podocyte biology has been the center of most research efforts in MCD over the last two decades (19–21). In fact, podocytes are key to maintain the integrity of the GFB as implied in forms of genetic nephrotic syndrome with abnormal or absent podocyte proteins and in knockout *in-vivo* models (19, 22–24). In MCD, there are changes in the expression, phosphorylation, and/or localization of podocyte-specific proteins such as synaptopodin and nephrin during relapse (25–27). In addition, the observations that some immunosuppressive drugs used in MCD may act directly on podocytes have also supported the concept of MCD as a podocyte disorder (28–32). In the recent years, there has been an increasing interest on the underpinning genetic architecture in MCD and several studies have identified gene variants that seem to confer susceptibility to the disease (33–36). Therefore, the pathogenesis of MCD seems to involve a complex interplay between immune cells, the glomerulus, and genetics (Figure 1). This complexity is reflected by the paucity of major breakthroughs in the understanding of the disease and lack of targeted therapies.

In this manuscript, we will provide an overview of the current experimental approaches available to study MCD and a review of candidate mediators and mechanisms of proteinuria involved in MCD and SSNS, that is commonly associated with MCD.

## Experimental Models of MCD

Table 1 shows a summary of current experimental models to study MCD. While these models have helped to advance, to some extent, our understanding of the disease, they still have significant limitations. Hence, there is an urgent need to develop better experimental approaches that mimic the natural course of human MCD.

**Table 1:** Overview of experimental models of MCD.

**Animal models (animal/route)**	**Strengths**	**Limitations**
PAN (Rat, IP)	- MCD-like injury - Acute and severe proteinuria	- Can evolve to FSGS - Direct toxicity on podocytes
LPS (Mouse, IP)	- MCD-like injury - Systemic immune activation - Acute proteinuria	- Mild proteinuria - Sepsis - Acute kidney injury
Poly: IC (Mouse, IP)	- MCD-like injury - Acute proteinuria - Mimic viral infection	- Mild proteinuria - Anecdotical use
Humanized mouse	- MCD-like injury - Incorporate PBMC from patients	- Mild proteinuria
	- Acute proteinuria	- Anecdotical use
Angptl4 (Rat, TG)	- MCD-like injury	- Slow onset proteinuria - Requires second insult (puromycin, etc.)
**Cell studies**	**Strengths**	**Limitations**
Human podocytes	- Well-characterized - Mechanistic control - High throughput - Relatively easy and inexpensive	- Does not recapitulate microenvironment - No shear stress - Limited glycocalyx - Inability to test permselectivity
Kidney organoids	- Incorporate different glomerular cells - Ability to test permselectivity and study crosstalk	- Requires expertise and longer timeline for experiments - Expensive - Does not include all glomerular cells - Limited throughput
3D co-cultures	- Ability to integrate two cell types and extracellular matrix - Microfluidic system - Reproduce shear stress - Ability to test permselectivity and study crosstalk - Develops glycocalyx	- Requires expertise and longer timeline for experiments - Expensive - Does not include all glomerular cells - Limited throughput

*PAN, puromycin aminonucleoside; IP, intraperitoneal; MCD, minimal change disease; FSGS, focal segmental glomerulosclerosis; LPS, lipopolysaccharide; Angptl4, angiopoietin-like 4; TG, transgenic. Poly: IC, Polyinosinic: polycytidylic acid*.

### Animal Models

Puromycin aminonucleoside (PAN) (rat model) and lipopolysaccharide (LPS) (mouse model) are the two most widely used models to induce sudden-onset proteinuria and MCD-like injury on kidney histology. The PAN model induces DNA damage via reactive oxygen species resulting in remarkable proteinuria, FPE, redistribution of proteins of the SD, changes in anionic charges in the glomerular basement membrane (GBM), and podocyte loss (37–41). In contrast, the LPS model is thought to directly activate the Toll-like receptor 4 (TLR-4) and downstream inflammatory pathways on podocytes resulting in mild and transient proteinuria, FPE, and changes in nephrin phosphorylation (20, 42, 43). Therefore, both models resemble some key features of MCD (sudden proteinuria, MCD-like injury on histology, and changes in podocyte proteins) (25–27), though apparently by different mechanisms. The main strength of the PAN model is the remarkable proteinuria and FPE, whereas that of the LPS model is the immune activation, likely relevant to MCD given its clinical association with infections (44, 45). However, both models have significant limitations. The PAN model often results in FSGS likely due to direct podocyte injury and loss rather than by a circulating factor (39). The LPS model is associated with a remarkable immune response resulting in sepsis, transient proteinuria, and AKI (46).

Other animal models have been described, but their use to study MCD is anecdotic. Polyinosinic: polycytidylic acid (Poly: IC) is a TLR-3 ligand that, when injected to mice, induces proteinuria and podocyte injury involving synaptopodin loss and FPE with no immune deposits on electron microscopy (47), mimicking features of MCD. Poly: IC is thought to mediate direct podocyte injury via TLR and activation of the inflammatory pathway nuclear factor-kappa B (NF-kB) (48). A weakness of this model is that proteinuria is mild and transient, but contrary to the LPS model, it is not associated with clinical sepsis (47). This model seems promising because it is well-tolerated by mice and results in glomerular changes that mimic MCD, but it remains to be determined whether variations from the original Poly: IC model of proteinuria may yield higher and/or sustained proteinuria like that observed in human MCD.

Sellier-Leclerc et al. developed a humanized mouse model of MCD by injecting CD34+ (a marker for hematopoietic stem cells) and CD34– peripheral blood mononuclear cells (PBMCs) from patients with MCD and FSGS into immunocompromised mice (49). Mice injected with CD34+ cells, but not with CD34–, developed albuminuria and partial FPE. Since CD34+ and CD34– induce the expansion of immature and mature T cells, respectively, authors postulated that proteinuria in this model could be mediated by immature T cells. The caveat of this model is that the degree of albuminuria was low and its use has not been reported since. It is unknown whether an additional insult (PAN, LPS, and Poly: IC) could have triggered a higher degree of proteinuria and still maintain MCD features.

A transgenic (TG) rat model characterized by the podocyte-specific overexpression of angiopoietin-like 4 (Angptl4) has also been associated to proteinuria and MCD-like changes on kidney histology (50). The main caveat of this model is the slow-onset proteinuria, contrary to the animal models described above and to the human disease. Therefore, these TG rats need to be exposed to an additional insult (puromycin, adriamycin, etc.) to accelerate proteinuria (50).

Another animal model of interest is the Buffalo/Mna rat. These rats spontaneously develop nephrotic syndrome with histological features of glomerulosclerosis. Notably, proteinuria and kidney lesions resolved when the Buffalo/Mna kidneys were transplanted into healthy rats, suggestive of a circulating factor(s) as driver of proteinuria (51). In addition, the Buffalo/Mna kidneys exhibited a greater infiltration of macrophages and T cells than control rats, along with an upregulation of macrophage and T-helper type 2 (Th2) cytokine transcripts before the progression of proteinuria (52). This model could serve to investigate potential mechanisms of disease progression and recurrence after transplantation.

### *In-vitro* Models

Cell culture studies are a valuable tool to study podocyte biology and they remain as standard approach to study mechanisms of disease in glomerulopathies. As such, different toxins (puromycin, LPS, and Poly: IC) and sera from patients with MCD have been used on cultured immortalized human podocytes in an attempt to replicate molecular changes triggered by the circulating factor(s) involved in MCD (43, 53). However, cultured human podocytes do not form secondary processes or slit diaphragms, show a variable expression of podocyte-specific proteins, and lack of cell-to-cell communication with glomerular endothelial cells (GEnC) and mesangial cells as in the human GFB (54). These are important limitations given the importance of the SD and the crosstalk between glomerular cells to maintain the integrity of the GFB. Another important consideration is that circulating toxins, at least those presumably present in the MCD sera or plasma, may not directly encounter podocytes in the human glomerulus as they do in culture systems.

To overcome the above limitations, new and promising *in-vitro* systems, such as kidney organoids and three-dimensional (3D) cocultures, have been developed over the last few years. These systems help to recapitulate the ontogeny of renal development and recapitulate features of the glomerular filtration barrier including cell-to-cell interactions and microfluid circulation. Proposed applications have included in-depth mechanistic studies and drug screening, gene knockouts and overexpression, and structural changes in response to sera of patient and various and sundry cytokines. These models have been reviewed in detail elsewhere and a detailed discussion is beyond the scope of this review (54, 55).

## Mediators and Mechanisms of Proteinuria in MCD

Table 2 shows a summary with some postulated mediators and mechanisms of proteinuria involved in MCD.

**Table 2:** Summary of candidate mediators and mechanisms of proteinuria in MCD.

**Candidate mediators**	**Potential mechanisms of podocyte injury**
**Circulating factors**	
*Cytokines*	
IL-13	- Loss and redistribution of podocyte proteins (60) - Glomerular CD80 upregulation (60)
IL-8	- Loss anionic charges in GBM (61)
IL-4	- Podocyte JAK signaling (62)
*Autoantibodies*	
UCHL1	- Unknown
Anti-nephrin	- Disrupting nephrin signaling
*Hemopexin*	- Loss anionic charges in GBM (77) - Disrupting nephrin signaling (78)
*Microbial products*	- Podocyte TLR activation and NFkB signaling (43, 47)
**Intraglomerular factors**	
IL-4	- Podocyte JAK signaling (62)
TNF-α	- Nephrin loss (88) - Podocyte FAK phosphorylation (89) - Glomerular CD80 upregulation (91) - NFkB activation (91) - Podocyte syndecan 4 shedding and β3 integrin signaling (104)
VEGF-A	- Dysregulated endothelial-podocyte crosstalk (92)
**Charge selective barrier**	
Angptl4	- Loss anionic charges in GBM (50)
Hemopexin	- Loss anionic charges in GBM (77)
IL-8	- Loss anionic charges in GBM (61)
**Podocyte dysfunction**	
CD80	- Prevent β1 integrin and Neph1 signaling (31, 111) - Activates local inflammatory pathways (47)
Angptl4	- Loss anionic charges in GBM (50) - Oxidative injury in GEnC (50) - Podocyte-endothelial crosstalk (50)
C-mip	- Disrupt nephrin signaling (121)
FAK	- Actin cytoskeleton reorganization (123) - Enhance metalloprotease activity (122)
ZHX1	- Podocyte angptl4 upregulation (124)
**Endothelial dysfunction**	
EG degradation and release of EG products (syndecans, etc.)	- Loss of electrostatic charges (101) - Podocyte activation via β3 integrin signaling (104)
CD80	- Activates local inflammatory pathways (47)
Caveolin-1	- Facilitates albumin transcytosis (133)

*IL, interleukin; GBM, glomerular basement membrane; UCHL1, ubiquitin carboxyl-terminal hydrolase L1; TLR, Toll-like receptor; TNF-α, tumor necrosis factor-α; VEGF, vascular endothelial growth factor; Angptl4, angiopoietin-like 4; GEnC, glomerular endothelial cell; FAK, focal adhesion kinase; ZHX1, zinc fingers and homeoboxes; EG, endothelial glycocalyx*.

## Circulating Factors

Since early 1970s, MCD has been thought to be mediated by some circulating factor(s). While this remains to be proven, there are some clinical and experimental observations that support this: (1) therapeutic response to immunosuppression, (2) lack of immune complexes in glomeruli, (3) resolution of proteinuria after transplanting kidneys with active MCD into patients without MCD, and (4) development of MCD-like injury in rats that received supernatants of PBMCs from patients with active SSNS and supernatants from T-cell hybridomas derived from MCD in relapse (4, 56–58). However, the clinical observations do not demonstrate causality and the experimental studies remain to be validated decades after the original publication (57, 58). Here, we will review some of the candidates circulating mediators and their postulated mechanisms of proteinuria in MCD.

### Circulating Cytokines

Several cytokines, predominantly from the Th2 subset, have been linked to MCD (59). A detailed review on systemic cytokine patterns in MCD is beyond the scope of this review. Here, we will specifically focus on those cytokines with a presumed pathogenic role in the development of proteinuria in MCD.

#### Interleukin-13 (IL-13)

The strongest evidence to support a role of IL-13 in MCD comes from a TG rat model characterized by high serum IL-13 levels (60). These rats developed nephrotic syndrome, FPE, loss and redistribution of some podocyte proteins, and CD80 upregulation in glomeruli, thereby mimicking some key features of human MCD (60).

#### Interleukin-8 (IL-8)

Garin et al. postulated that systemic IL-8 could play a role in MCD. Rats infused with IL-8, reaching serum levels similar to those observed in MCD, developed proteinuria due to an increased metabolism of glycosaminoglycans (GAGs) in the GBM, mimicking the anionic loss reported in the GBM of some patients with MCD (61). Nevertheless, IL-8 only caused mild proteinuria.

#### Interleukin-4 (IL-4)

High systemic IL-4 via liver overexpression caused proteinuria and podocyte FPE in mice, which was ameliorated with a JAK inhibitor, suggesting that IL-4 may mediate proteinuria by activating JAK signaling in podocytes (62). In a single-center study, 10 of 29 patients with active MCD had a positive staining for phosphorylated STAT6, a surrogate marker of IL-4 signaling in glomeruli, whereas it was positive in only 1 of 23 controls.

There is some evidence that IL-13, IL-8, and IL-4 could play a role in experimental models of proteinuria, but the significance of these studies remains unclear due to the lack of further validation by other research groups and by the heterogenous pattern of these cytokines in patients with MCD (53, 59, 63–65).

While T-cell effectors are the source of the above and other pro-inflammatory cytokines, a deficiency in regulatory T cells (Treg cells) has also been implicated in the pathogenesis of MCD (14, 66). An example is the association of MCD with immune dysregulation, polyendocrinopathy, enteropathy, and X-linked (IPEX) syndrome. This is an immunodeficiency syndrome characterized by a FOXP3 mutation that inactivates Treg cells (67). In addition, Treg cells express CTLA-4. This is an important modulator of the immune response by binding to CD80 on antigen presenting cells (APCs). Of note, patients with MCD have a high CD80/CTLA-4 ratio in urine, suggesting that an imbalance in these molecules may have a role in MCD (68).

In summary, experimental studies on animal models have shown a possible role for some cytokines in the pathogenesis of proteinuria. However, the results of these studies have not been confirmed in human disease. This may be due to the variable methodology among studies, the heterogeneity of the disease, the differences between human MCD and animal models, and the complex interplay among pro- and anti-inflammatory cytokines, immune cells, and glomerulus. To date, there is no evidence that a single cytokine is a key mediator of podocyte injury in MCD.

### B Cells and Autoantibodies

While most studies have focused on T-cell-related cytokines, the efficacy of anti-CD20 therapy [rituximab (RTX)] to reduce the frequency of relapses in SSNS and MCD has brought an increasing interest on the potential role of B cells in the disease pathogenesis (69). It remains unclear whether B cells may contribute to the disease by promoting certain T-cell responses or by releasing autoantibodies against podocyte proteins (70–72). For instance, Oniszczuk et al. found higher circulating levels of plasmablasts and B-cell activating factor in the serum of adults with MCD during relapse (73), but whether these drive T-cell activation in MCD remains to be determined. Colucci et al. found that patients with idiopathic nephrotic syndrome (INS) and poor response to therapies carry higher levels of T cells with hyposialylated immunoglobulin M (IgM) on the surface (72). Supernatants from these T cells caused podocyte cytoskeletal rearrangements *in vitro*, but this was prevented when T cells were incubated with sialylated IgM, suggesting that IgM on the T-cell surface modulates T-cell responses and that B- and T-cell crosstalk may play an important role in the pathogenesis of MCD (72). Trachtman et al. recently showed that IgM can trigger the classical pathway of complement in glomeruli from patients with INS (74). These findings emphasize the potential pathogenic role of B cells in nephrotic syndrome.

Furthermore, B cells produce antibodies. Thus far, there have been two candidate autoantibodies proposed as mediators of MCD, but the lack of immune complexes on kidney tissue remains an argument against a key pathogenic role of these autoantibodies.

#### Antiubiquitin Carboxyl-Terminal Hydrolase L1 (UCHL1) Antibodies

Combining human, experimental, and animal studies, Jamin et al. found elevated anti-UCHL1 antibody titers in plasma in about half of children with SSNS in relapse compared to controls (71). These autoantibodies targeted podocytes causing cell detachment *in vitro* and proteinuria and MCD-like injury *in vivo*. Interestingly, antibody titers were not increased in adults with active MCD (71). If these findings are validated, this would support a pathogenic role of autoantibodies and suggest that childhood- and adulthood-onset MCD may have a different underlying pathophysiology.

#### Antinephrin Antibodies

Watts et al. recently found circulating antinephrin antibodies in 29% of patients with active MCD [“Autoantibodies against nephrin elucidate a novel autoimmune phenomenon in proteinuric kidney disease.” Medrxiv (Preprint). Available at https://www.medrxiv.org/ content/10.1101/2021.02.26.21251569v1.full]. In the MCD glomerulus, authors showed granular immunoglobulin G (IgG) deposits colocalizing specifically with nephrin, but not with other podocyte proteins. This is an attractive finding as antinephrin antibodies mediate recurrence of nephrotic syndrome in patients with congenital nephrotic syndrome. Watts et al. postulated that circulating antinephrin antibodies may bind to nephrin changing its localization in the slit diaphragm, thereby resulting in proteinuria. However, it remains unknown whether these antibodies play a causative role either as primary or secondary insult to the podocyte or whether they may represent a paraphenomenon. Of note, circulating antinephrin antibodies are also present in patients with diabetes, but they were not associated to a higher risk of proteinuria (75). While antinephrin antibodies cause proteinuria in rats, they only induce a partial retraction of podocytes; so, the exact mechanisms by which these autoantibodies may cause proteinuria remains unclear (76).

Future studies are needed to determine the pathogenic role of B cells, to assess for causality between circulating autoantibodies and proteinuria in MCD, and to screen for novel candidate autoantibodies.

### Hemopexin

Hemopexin is a plasma glycoprotein with high affinity for heme and immunoregulatory properties. When infused into rats, hemopexin caused reversible proteinuria and podocyte FPE resembling MCD-like injury (77). Mechanistically, it is thought to contribute to proteinuria by reducing anionic charges in the GBM *in vivo* and by disrupting nephrin signaling *in vitro* (77, 78). Patients with MCD may carry an “active” form of hemopexin. While plasma levels were first reported low in patients with MCD compared to controls, a recent study found that serum and urine levels of hemopexin are high in children with active nephrotic syndrome (79, 80); so, larger studies by using standardized methods to quantify hemopexin could provide insights into the value of hemopexin as marker or mediator of disease. More recently, plasma hemopexin was found to discriminate among patients with SSNS and steroid-resistant nephrotic syndrome (SRNS) with lower levels noted in SSNS compared to SRNS (81).

### Microbial Products

In children with MCD, proteinuria is often triggered by infections (44). In mice, TLR ligands induce transient proteinuria and podocyte injury. However, it remains unknown whether viral or bacterial products directly stimulate TLR on podocytes or whether proteinuria may be the result of an exaggerated systemic immune response in susceptible patients (42, 47).

### Others

Over the last few years, proteomic and metabolic studies have identified candidate biomarkers in urine or plasma (α1-macroglobulin, adiponectin, etc.) to discriminate MCD from FSGS and SSNS from SRNS, but whether some of these may also have a pathogenic role in proteinuria is still unclear (81, 82). In 2021, there have been numerous cases reported of new onset or relapsing MCD following COVID-19 vaccine (83). These cases shared a strong temporal association between vaccine administration and onset of proteinuria, highly suggestive of an exaggerated and rapid T-cell-mediated immune response to viral messenger RNA (mRNA). Future studies are required to investigate a potential causal link between the COVID vaccines and MCD. On the other hand, Angeletti et al. recently reported that protein-based vaccines are not associated with a higher risk of relapse in patients with nephrotic syndrome (84).

## Local (Intraglomerular) Factors

Most researchers have historically focused on the study of circulating cytokines, but recent studies have recognized the importance of the local microenvironment in the development of podocyte injury in the experimental models. Here, we will review some of the molecules, thought to be released within the glomerulus, that have been linked to the pathogenesis of MCD.

### Interleukin-4

In an elegant study, Kim et al. planted a B-cell antigen (hen egg lysozyme) on the GBM and found that injection of polarized antigen-specific B cells led to transient proteinuria within 24 h and histological changes consistent with MCD-like injury. In contrast, transfer of polarized antigen-specific B cells that were IL-4 deficient did not cause proteinuria, suggesting that proteinuria was mediated by local activated B cells and release of IL-4 (62). Interestingly, circulating IL-4 was undetectable, suggesting that the local release of IL-4 was sufficient to induce proteinuria and podocyte injury *in vivo* (62).

### Tumor Necrosis Factor-α (TNF-α)

Studies involving TNF-α have largely focused on FSGS rather than MCD. Because some patients with MCD eventually develop FSGS, intraglomerular TNF-α remains a target of interest as mediator of podocyte injury in MCD (85, 86). Sera from patients with FSGS are able to increase TNF-α expression in cultured human podocytes and consistent with this, glomerular TNF-α is increased in patients with FSGS (86, 87). While glomerular TNF-α is inversely correlated with estimated glomerular filtration rate (eGFR), it did not show a correlation with serum TNF-α, reflecting the discrepancy between the systemic and glomerular cytokine levels (86). Mechanistically, TNF-α downregulates nephrin expression and phosphorylates paxillin and focal adhesion kinase (FAK) leading to cytoskeletal rearrangement (88, 89). This may be relevant to MCD, as podocyte FAK is activated in these patients (90). In addition, TNF-α activates the inflammatory NF-kB pathway and can induce CD80 expression on podocytes (see CD80 section) (91).

### Vascular Endothelial Growth Factor (VEGF)

Podocytes are a source of several factors that act as ligands of receptors expressed by GEnC and this cellular crosstalk is key to maintaining endothelial homeostasis and the integrity of the GFB as demonstrated by landmark studies in the field (92). VEGF-A is the most well-characterized molecule of the VEGF family and it has been implicated in the pathogenesis of diabetic nephropathy, preeclampsia, and thrombotic microangiopathy (92). Podocyte-specific loss of VEGF-A in mice prevents glomerular development and formation of glomerular endothelium and the inactivation of single VEGF-A allele leads to endothelial injury and end-stage renal disease. In contrast, the podocyte-specific overexpression of VEGF_164_ results in collapsing glomerulosclerosis, suggesting that VEGF expression within the glomerulus is tightly regulated (92). In MCD, glomerular expression of VEGF has been reported high during relapse by some authors but not by others (93, 94). The discrepancy between studies could be related to methodology and the heterogeneity of the disease. Another consideration is that a kidney biopsy only reflects the molecular signature at a specific time point, whereas VEGF expression may fluctuate during different stages of relapse and remission.

### Others

Several molecules involved in endothelial–podocyte crosstalk such as angiopoietins 1 and 2 and VEGF-C have been linked to proteinuric glomerular diseases such as preeclampsia and diabetic nephropathy, but their role in MCD remains to be investigated.

Further studies are needed to characterize the cytokine and inflammatory signature in the MCD glomerulus, the cellular source of these local cytokines (infiltrating T and/or B cells, podocytes, other glomerular cells, etc.) and the stimuli triggering such cell responses. Likewise, a reduced number of Treg cells has been reported in the MCD glomerulus (95), so it is possible that the imbalance between effectors T and Treg cells may be important to determine a pro- *vs*. anti-inflammatory microenvironment in the glomerulus.

## Role of the GFB as a Charge Selective Barrier in MCD

The GFB is a size and charge-selective functional unit that allows the free flow of water and small molecules while preventing the passage of plasma proteins into urine. It consists of three layers: GEnC and associated glycocalyx, the GBM and podocytes with their foot processes, SD, and glycocalyx. The disruption of the GFB at any layer can result in proteinuria, but podocytes play a critical role in the formation and integrity of the GFB (96). However, the cause of proteinuria in MCD was historically attributed to the loss of negative charges in the GBM based on the observation that anionic charges were reduced in the GBM in animal models of podocyte injury and in some patients with MCD (40, 97). Subsequent experimental models failed to demonstrate a causal link between the GBM charges and proteinuria *in vivo* (98), so this theory was nearly abandoned in the twenty first century. More recently, Huizing et al. found evidence of glomerular hyposialytation in 26% of patients with proteinuric glomerular disease including MCD and other glomerular diseases (99). These observations provided the rationale for an ongoing randomized trial to test whether N-acetylmannosamine (ManNAc), a sialic acid precursor, may ameliorate proteinuria in human glomerular disease (99). Further studies are warranted to identify the primary affected glomerular cell/protein with hyposialytation, the triggering insult, and whether ManNAc restores sialylation and ameliorates proteinuria in human MCD.

The GBM and, to a lesser extent, podocytes have been the focus of interest for researchers studying the role of charges in MCD, but it is notable that little attention has been paid to the endothelial glycocalyx (EG). This is a thick meshwork of GAG and proteoglycans negatively charged that covers the entire endothelium and its fenestrations. It has been postulated that the EG is a major site for the generation of an electrokinetic field that prevents the passage of negatively-charged plasma proteins such as albumin (100, 101). In support to this, the loss of EG is associated to proteinuria in experimental models of diabetes and sepsis and human diseases (102). Of note, a study found an increase in circulating syndecan 1, as surrogate of EG degradation, in patients with MCD (103). So, further studies need to address whether the EG may be relevant for the development of proteinuria in MCD. It is possible that the EG injury favors the passage of albumin through the GFB and that EG products may modulate biological processes including podocyte response to injury. For example, syndecan 4, an important component of the EG, can activate podocyte via TRCPC6 and β3 integrin signaling (104).

## Podocyte Dysfunction

In MCD, podocytes experience alterations in shape known as FPE and changes in the regulation of key proteins such as nephrin and synaptopodin (25–27). These changes include nephrin downregulation, redistribution, dephosphorylation, and synaptopodin downregulation (25–27). However, these molecular dysregulations are not specific of MCD and it is still unclear whether they are the cause of the podocyte injury and/or FPE or whether they represent a non-specific adaptive response of podocytes to injury. Indeed, MCD has been associated with an increased in podocyte autophagic activity (105, 106) that is an important mechanism for stress adaptation. Notably, progression from MCD to FSGS on histology was associated with a decline of autophagic activity (106).

Podocytes are key to prevent proteinuria, but there is a poor correlation between the level of proteinuria and FPE in patients with MCD and other glomerular diseases (107), questioning a direct cause and effect between FPE and proteinuria and suggesting that other cell types may contribute to the disease.

### Candidate Mediators of Proteinuria in Experimental MCD

Current *in-vivo* approaches to study MCD (PAN and LPS) result in a reduction and/or redistribution of podocyte proteins mimicking some key features of human MCD. Here, we will review some of the most studied and/or promising candidate mediators of podocyte injury in experimental models of MCD.

#### CD80

This is a costimulatory molecule expressed by APCs upon activation. In cultured podocytes, CD80 expression is upregulated upon injury with LPS and TNF-α and mediates actin rearrangements, a surrogate marker for FPE (42, 91). There is indirect evidence *in-vivo* supporting a role of CD80 as mediator of proteinuria in the LPS model. CD80 knockout mice do not develop proteinuria following LPS exposure and CD80 expression on non-hematopoietic cells is also necessary for TLR stimulation to cause albuminuria in mice (42, 91). Also, CD80 is excreted into urine following TLR stimulation (47). However, some groups could not identify CD80 expression in podocytes following LPS questioning the role of CD80 in proteinuria (108). More recently, we and others demonstrated that CD80 is upregulated by kidney endothelial cells following LPS by using immunofluorescence and endothelial-specific translating ribosome affinity purification (EC-TRAP) and high-throughput RNA sequencing analysis, respectively (109, 110). Mechanistically, CD80 is thought to prevent talin binding to β1 integrin and its downstream signaling and to prevent Neph1 binding to nephrin, thereby altering actin polymerization and organization (31, 111).

In humans, CD80 was initially found upregulated in podocytes in some patients with MCD during relapse. However, there have been contrasting results among studies questioning the validity of the CD80 staining in human kidney tissue and this has tamped down the initial enthusiasm for this molecular target (108, 112). Our group recently demonstrated that CD80 is indeed present in podocytes in MCD, but, surprisingly, most CD80 was lining the capillary lumens in an endothelial pattern (109). We and others also showed that urinary CD80 levels are consistently high in a subset of patients with MCD in relapse compared to controls and to patients with other proteinuric glomerular disease (68, 112–115). Interestingly, there are two cases reported in that patients with active MCD and high CD80 in urine underwent rapid transient and sustained remission following anti-CD80 therapy, respectively (116, 117). In contrast, the efficacy of anti-CD80 therapy in FSGS, usually associated to normal CD80 levels in urine, remains controversial (118). These observations suggest a potential link between CD80 or downstream pathways and proteinuria in selected patients with MCD (those with either high urinary or glomerular CD80).

#### Angiopoietin-Like 4

This is a glycoprotein highly expressed by the liver and adipose tissue. Podocyte-specific Angptl4 overexpression in rats caused albuminuria and FPE over time without immune complex deposition mimicking features of MCD. Proteinuria was exacerbated when these transgenic rats received a single dose of puromycin and it was partially ameliorated when animal received steroids or ManNAc, which is a sialic acid precursor (50). In this model, injured podocytes released hyposialylated Angptl4 that bound to the GBM neutralizing its negative charges and also enhanced oxidative injury to GEnC *in vitro*. In contrast, normosialylated Angptl4 is released into circulation and this mediates hyperlipidemia and ameliorates proteinuria by interacting with glomerular endothelial αvβ5 integrin in different animal models of proteinuria including FSGS and diabetic nephropathy (50, 119). Still, the mechanisms of proteinuria in the podocyte Angptl4 model are not fully understood. In humans, Angptl4 was found overexpressed in podocytes from few patients with MCD in relapse (50), but these findings were not validated in a larger clinical study (120). Angptl4 is excreted into urine at high levels in different proteinuric diseases, suggesting that Angptl4 may reflect a non-specific response of podocytes to various insults rather than being a specific marker or mediator of MCD (120). More clinical studies are necessary to determine whether Angptl-4 may contribute to human MCD.

#### c-mip

This was initially found elevated in T cells of patients with MCD in relapse and subsequently in podocytes of patients with active MCD and other forms of proteinuric glomerular disease. In animal studies, c-mip overexpression in podocytes causes albuminuria, FPE, and loss of total and phosphorylated nephrin without evidence of inflammatory changes in the glomerulus, thereby mimicking key features of MCD (121). LPS triggers podocyte c-mip upregulation and this seems independent of the cellular or humoral immunity. Mechanistically, c-mip interferes with Fyn binding to nephrin preventing downstream signaling and nephrin phosphorylation, which is a critical for podocyte restoration following transient podocyte injury (121). To date, there is strong experimental data to support a potential role of c-mip as mediator of proteinuria in MCD and other glomerular disease, but further clinical studies are necessary.

#### Focal Adhesion Kinase

FAK is a non-receptor tyrosine kinase that resides at sites of integrin clustering serving as a link between the extracellular matrix and the acting cytoskeleton. Upon phosphorylation, FAK modulates cell motility and migration and also contributes to the secretion of matrix metalloproteinases (122). In podocytes, FAK activation is necessary for the development of proteinuria and FPE following LPS (123). In experimental models, nephrin phosphorylation is an upstream activator of the Cas-Crk pathway, involving FAK activation (90). In patients with MCD, but not FSGS, FAK activation is observed in podocytes during relapse. However, MCD is associated with a reduction in nephrin phosphorylation (26), so that the upstream signaling triggering FAK activation in MCD is not fully understood.

#### Zinc Fingers and Homeoboxes (ZHX)

This refers to a family of transcriptional factors (ZHX1, ZHX2, and ZHX3) that regulates the expression of key podocyte genes. ZHX proteins localize at the membrane as hetero- or homodimers. Using different injury models of proteinuria, Macé et al. showed that translocation of ZHX proteins from the membrane into the nucleus may result in distinct types of nephrotic syndrome (124). Nuclear ZHX3 alone or in combination with ZHX2 was associated to FSGS, whereas nuclear ZHX1 was associated to MCD-like injury and podocyte Anpgtl4 upregulation in culture systems. Thus, this study provided a mechanism by which podocyte Angptl4 expression may be regulated in MCD and it proposed a plausible pathway that may be involved in the development of several forms of proteinuric kidney disease (124). Data on human MCD are still scarce. The same group found an increase in ZHX1 expression in podocyte nuclei from patients with active MCD, whereas ZHX2 expression was downregulated compared to controls (124).

In summary, there are several candidate mediators of podocyte injury and proteinuria in models of MCD. Nevertheless, the lack of specificity to discriminate among models of proteinuria and the lack of validation in large clinical studies do question their clinical relevance for human MCD. This emphasizes the urgent need to develop novel experimental models and/or approaches to study MCD.

### Candidate Targets Identified in Human Tissue

The use of novel approaches such as transcriptomics has helped to identify molecules and pathways relevant for the pathogenesis of the human disease. Sanchez-Niño et al. showed higher expression of fibroblast growth factor-inducible 14 (Fn14), monocyte chemotactic peptide-1 (MCP-1), and NF-kB in podocytes from kidney tissue of patients with FSGS, but not from MCD (125). Bennet et al. reported an increased expression of genes involved in inflammation and fibrosis (osteopontin, CD24, CCL3, CXCL2, CXCL14, SOX9, etc.) in FSGS glomeruli compared to controls. Likewise, authors found a decrease in podocyte-specific genes (NPHS1, WT1, VEGF, etc.) in patients with FSGS compared to controls (126). Hodgin et al. identified few differentially expressed genes in glomeruli from MCD in relapse *vs*. controls. Specifically, these genes are involved in amino acid and metabolic processes (BHMT, DDC, and XPNEP2) and cell adhesion (CDH11, MPZL2, OPCML, and TRO) (127). Using single cell transcriptomics, Menon et al. demonstrated an upregulation of alpha-2 macroglobulin in GEnC from patients with FSGS compared to living donors and this was associated to poor clinical outcomes (128).

These novel approaches and collaborative efforts are key to elucidate the different molecular signatures linked to MCD. In particular, single cell transcriptomics is important to identify the dysregulated cell type within the glomerulus. Likewise, future research should also address the upstream pathways leading to these intraglomerular changes.

## Endothelial Dysfunction

Endothelial cells line the entire vasculature and, in the glomerulus, are in close proximity to podocytes. Endothelial–podocyte communication is critical for the maintenance of the GFB. In addition, endothelial cells also have the machinery to present and process antigens and are important modulators of the immune response and inflammation (129). These features along with the assumption of a circulating factor involved in MCD could make endothelial cells an attractive cell target for the disease pathogenesis. However, studies involving GEnC in MCD are anecdotical unlike in other glomerular diseases. There have been few reports that showed evidence of endothelial dysfunction, ultrastructural changes in the glomerular endothelium, and upregulation of markers of cell activation, such as CD80 and caveolin-1, in GEnC of patients with MCD during relapse (103, 109, 130–132). These findings suggest that the endothelium is injured and/or activated in MCD and raises the possibility that activated GEnC may contribute to a pro-inflammatory milieu in the glomerulus rather than being an innocent bystander. For instance, CD80 is associated with activation of downstream inflammatory pathways and caveolin-1 mediates albumin transcytosis and endothelial cell function (133). More recently, Trachtman et al. showed that IgM can bind to epitopes on injured GEnC and activate the complement pathway (74). In addition, injury to the endothelium may facilitate the passage of proteins through the GFB due to the loss of the glycocalyx that serves as charge barrier (101). Therefore, further studies are warranted to determine whether activated GEnC plays a pathogenic role in proteinuria in MCD.

## Genetics

Epidemiological studies have shown cases of familial SSNS and certain ethnic differences across populations. For instance, SSNS is more common in Asian children and it may have a more difficult clinical course in patients of African-American or Hispanic descents (134). However, Mendelian mutations have been rarely described in SSNS or MCD contrary to that observed in SRNS or FSGS. These findings suggest that SSNS and MCD are polygenic diseases with a complex inheritance pattern influenced by individual genetic risk and environmental factors. Given the association of SSNS and MCD with immune dysregulation, HLA genes have been a target of interest for researchers (33, 135). Numerous groups used an HLA candidate approach to identify genetic risk loci for SSNS and identified several variants including HLA-DQB, HLA-DBA, HLA-DRB1, HLA-DQB1, HLA-DQW2, and HLA-DR7 (134). Using a non-biased approach such as genome-wide association studies (GWASs), Gbadegesin et al. identified HLA-DQA1 and PLCG2 missense variants as candidate risk loci for children with SSNS. HLA-DQ1 variants resulted in perturbation of protein secondary structure, which may alter the process of antigen presentation in these patients. Notably, HLA-DQA1 is also reported in IgA nephropathy and membranous nephropathy, suggesting a shared immune dysregulation among these proteinuric diseases (36). As previously mentioned, IL-4 and IL-13 have been linked to MCD/SSNS. Several studies have investigated the potential association between gene variants and MCD with contrasting results. Al Rushood et al. found no association between IL-4 and IL-13 gene polymorphisms and susceptibility to SSNS (136), whereas Acharya et al. reported a possible association of these gene variants and MCD (137). In a similar study, Ikeuchi et al. showed an association between STAT6 gene polymorphisms and MCD (138) contrary to that reported by others (137). Using GWAS, Jia et al. recently identified NPHS1 (nephrin) and TNFSF15 regions as susceptibility factors for childhood SSNS (139).

Genetics studies continue to shed light into potential targets and pathways relevant for the disease and also reinforce the role of immunity in MCD. Nevertheless, these studies demonstrate associations rather than causality.

## Conclusion

Minimal change disease is a clinical–histopathological entity with variable clinical outcomes. Despite research efforts, the mechanisms of proteinuria remain poorly understood and this has hampered the development of targeted therapies. MCD involves a complex interplay between environmental factors, genetic susceptibility, immune dysregulation, and the glomerular microenvironment, suggesting that MCD is not simply an immune or podocyte disease. This may explain the heterogeneity of the human disease, which together with the lack of good animal models, remain major obstacles to elucidate the pathogenesis of MCD. Therefore, future research strategies should integrate analysis of timed human biosamples including large and well-characterized cohort of patients along with novel experimental models including animal studies and state-of-the-art *in-vitro* approaches to improve our understanding of cell-to-cell interactions in the disease.

## Author Contributions

SP, FP, and GC-F contributed to the design of the review paper. FP created figures and tables. All authors contributed to the revision and reading of the manuscript and approved the submitted manuscript.

## Conflict of Interest

The authors declare that the research was conducted in the absence of any commercial or financial relationships that could be construed as a potential conflict of interest.

## Publisher's Note

All claims expressed in this article are solely those of the authors and do not necessarily represent those of their affiliated organizations, or those of the publisher, the editors and the reviewers. Any product that may be evaluated in this article, or claim that may be made by its manufacturer, is not guaranteed or endorsed by the publisher.
